# Systematic inclusion of mandatory interprofessional education in health professions curricula at Gunma University: a report of student self-assessment in a nine-year implementation

**DOI:** 10.1186/1478-4491-7-60

**Published:** 2009-07-23

**Authors:** Hatsue Ogawara, Tomoko Hayashi, Yasuyoshi Asakawa, Kiyotaka Iwasaki, Tamiko Matsuda, Yumiko Abe, Fusae Tozato, Takatoshi Makino, Misako Koizumi, Takako Yasukawa, Hideomi Watanabe

**Affiliations:** 1 Department of Laboratory Sciences, School of Health Sciences, Gunma University, Gunma, Japan; 2 Department of Nursing, School of Health Sciences, Gunma University, Gunma, Japan; 3 Department of Physical Therapy, School of Health Sciences, Gunma University, Gunma, Japan; 4 Department of Occupational Therapy, School of Health Sciences, Gunma University, Gunma, Japan; 5 Department of Internal Medicine, Seirei Hamamatsu General Hospital, Shizuoka, Japan

## Abstract

**Background:**

The mandatory interprofessional education programme at Gunma University was initiated in 1999. This paper is a statistical evaluation of the programme from 1999 to 2007.

**Methods:**

A questionnaire of 10 items to assess the achievement levels of the programme, which was developed independently of other assessment systems published previously, was distributed, as well as two or three open-ended questions to be answered at the end of each annual module. A multivariate analysis of variance model was used, and the factor analysis of the responses was performed with varimax rotation.

**Results:**

Over all, 1418 respondents of a possible 1629 students completed the survey, for a total response rate of 87.1%. Cronbach's alpha of 10 items was 0.793, revealing high internal consistency. Our original questionnaire was categorized into four subscales as follows: "Role and responsibilities", "Teamwork and collaboration", "Structure and function of training facilities", and "Professional identity". Students in the Department of Occupational Therapy reached a relatively lower level of achievement. In the replies to the open-ended questions, requests for the participation of the medical students were repeated throughout the evaluation period.

**Conclusion:**

The present four subscales measure "understanding", and may take into account the development of interprofessional education programmes with clinical training in various facilities. The content and quality of clinical training subjects may be remarkably dependent on training facilities, suggesting the importance of full consultation mechanisms in the local network with the relevant educational institutes for medicine, health care and welfare.

## Background

In 1988, the World Health Organization (WHO) identified multiprofessional education as the process by which students and practitioners from various health professions learn together with the goals of interaction and collaboration in providing health promotion, disease prevention, curative services, rehabilitation and palliation [1]. A recent report from a nurse-coordinated, multidisciplinary, family-based, ambulatory programme (EUROACTION) demonstrated that healthier lifestyles and improvement in risk factors were achieved among patients with coronary heart disease and those at high risk for cardiovascular disease and their partners as compared to standard care, indicating the usefulness of interprofessional working (IPW) [2].

Interprofessional education (IPE) plays an important role in the acquisition of an attitude for IPW in graduate and undergraduate students [3]. Recently there has been an explosion of interest in IPE on the part of academic institutions around the world [4]. To plan and perform IPE activities effectively, the characteristic culture of the country as well as the norms, strengths and constraints of the academic institutes must be taken into account. Unique IPE must be adapted for each educational setting in each country.

In Gunma University, the School of Health Sciences was incorporated from the junior college department into the Faculty of Medicine in 1996. For the faculty integration, one of the most important aspects of education has been IPE. A few years were spent in preparing for the educational system development, and the original and distinctive IPE was delivered to the third year of undergraduate students for the first time in 1999.

The mandatory IPE practice-training curriculum, designated as "Simulated interprofessional training among students of different professions in health sciences", has been implemented continuously for over 10 years. A systemic assessment for the achievement was developed independently of those described elsewhere [5,6], and we reviewed the effectiveness of the curriculum from a team-building point of view. The present IPE programme has thus far been planned, implemented and evaluated independently of scientific research.

The present educational programme has been approved as a "Support Programme for Distinctive University Education" by the Ministry of Education, Culture, Sports, Science and Technology (MEXT). With this financial support, in the present paper, the effectiveness and the limitations were elucidated statistically using our own assessment measures, and will be discussed in comparison with the literature.

## Methods

### GUSHS curriculum guidelines

Gunma University School of Health Sciences (GUSHS), which provides interprofessional curricula for students majoring in nursing (NS, 80 students), laboratory sciences (LS, 40 students), physical therapy (PT, 20 students) and occupational therapy (OT, 20 students), was upgraded from the junior college department and incorporated into the Faculty of Medicine in 1996.

The IPE programme in GUSHS consists of two types of subjects. One type is a lecture style, which includes two subjects delivering information to first-year students and teaches the details and value of IPW. These lecture-style subjects are "Holistic Medicine/Teamwork Studies", a mandatory basic science, provided in the first term, and "Interprofessional Work Overview", an elective course in general education in the latter term, totalling 15 lessons for each subject. Another is a training-based subject called Teamwork Training. Building on the professional expertise acquired in the second year, third-year students participate in this mandatory training subject, a core programme of our IPE. The third-year students also learn clinical skills. In both third- and fourth-year students, clinical training subjects and research for a graduation thesis are provided. Thus, the GUSHS provides comprehensive health professions education curricula that are well balanced between specialized clinical education and holistic medical approaches.

### Contents of the subject "Teamwork Training"

**Figure 1:**
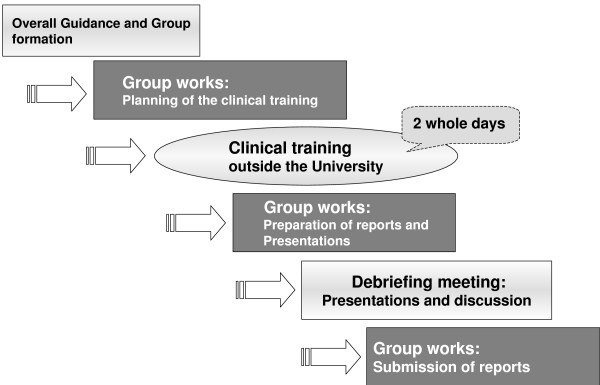
**Flowchart of IPE implementation in GUSHS**.

This IPE training, consisting of 45 two-hour lessons conducted throughout the third academic year, has been provided for 10 years, since the 1999 academic year. The flowchart of IPE in GUSHS is shown in Figure 1. After a short orientation, teams of students from different departments were allocated to the training facilities through games.

The design of the training agenda and planning of the clinical training were developed through several group consultations. Clinical training was then implemented outside the university at the assigned training facilities for two full days.

Approximately 20 facilities were asked to cooperate with the clinical training outside the university, selected from the following seven fields: "Hospital Medicine", "Community Health Care", "Care at Home", "Rehabilitation", "Medical Care for the Mentally Ill", "Paediatric Care" and "Elder Care". These facilities have played a major role in delivering diversified health care services.

After this clinical training, several group consultations were held to prepare a report on achievements and lessons learnt, which were presented at a debriefing meeting. The debriefing meeting of the "Teamwork Training" course was held, consisting of each group's presentation and general discussions, and then several group consultations were held to prepare clinical training reports for a brief report book.

The management body, designated as the Interprofessional Education Committee of Gunma University (IPEC-GU) consists of two professors from GUSHS and four associate professors. The Committee contacts the selected training facilities, prepares an annual plan, organizes preparatory and management meetings, supervises academic staff who implement training with students, establishes standards for evaluation, conducts and analyses the "post-training evaluation surveys" and compiles reports on training.

Approximately 20 academic staff selected from the four departments facilitated the students' training groups according to the educational guidelines. A training group consists of eight students: four from the Department of Nursing, two from Laboratory Sciences, and one each from Physical Therapy and Occupational Therapy, which is proportional to the enrolment capacity of each department.

### Assessments of the IPE programme by students

To assess achievement levels of IPE in GUSHS, a questionnaire survey was conducted on 1629 undergraduate students during the nine years from 1999 to 2007, and 1418 respondents completed the survey. The numbers of respondents were as follows: nursing (N, 690), laboratory sciences (LS, 373), physical and occupational therapies (PT, 180; OT, 175. Students were asked to complete a short questionnaire with the following 10 items:

1. organization of the facility

2. function of the facility

3. roles of each profession in the facility

4. operations and tasks of each profession in the facility

5. collaboration among professionals working in the facility

6. your profession's role and uniqueness

7. teamwork experienced in the training facility

8. membership and leadership in group activities

9. teamwork required in various fields

10. importance of teamwork.

Students rated each question on a four-point scale of understanding, ranging from; "I fully understood" (4), "I understood"(3), "I did not understand well" (2), and "I did not understand at all"(1) – two positive and two negative responses, respectively. Two or three open-ended opinions at the end of each IPE module were also obtained.

### Statistical analysis

A multivariate analysis of variance (MANOVA) model was used, and then factor analysis of the responses was performed with varimax rotation by means of the Statistical Package for the Social Sciences (SPSS, version 16.0J). This procedure was used both to reduce a large dataset and to identify clustering items in the scale. Scrutiny of the clustered items enabled hypothetical inferences to be made about relationships between variables. Measures of internal consistency (coefficient alpha) of subscales and items were obtained using standard psychometric evaluation procedures. Significance levels were set at *p *values less than 0.05.

## Results

Overall, 1418 respondents of a possible 1629 completed the survey, for a total response rate of 87.1%. Cronbach's alpha of 10 items was 0.793, revealing a high rate of internal consistency.

### Factor analysis of the responses

The "Department belonged to" was added to the 10 items of the questionnaire as another item, and points from one to four were given for each department in descending order according to the mean score of total questions in the department; i.e., 1 for Department of Nursing (mean score of 3.440), 2 for Laboratory Sciences (3.393), 3 for Physical Therapy (3.366), and 4 for Occupational Therapy (3.259). The factors were examined after varimax rotation. Items with loadings less than 0.4 and those with loadings over 0.4 that appeared in more than one factor were discarded. As a result, one item, Q7-"Teamwork experienced in the training facility", showing factors loading of higher than 0.4 in two subscales, was removed. By factor analysis, four subscales were obtained, as shown in Table 1. In the four subscales, three corresponded well to those initially named "Roles and responsibilities", "Teamwork and collaboration", and "professional identity", as described by Parcell and Bligh [5], and another – "Structure and function of training facilities" – was new in the present study.

**Table 1:** Summary of factor analysis contributing to each subscale*

		**Subscale**			
		
**Question number**	**Items**	1	2	3	4
3	Role of each profession in the facility	**0.781**	0.115	0.173	0.087
4	Operations and tasks of each profession in the facility	**0.621**	0.193	0.165	0.086
5	Collaboration among professionals working in the facility	**0.465**	0.283	0.139	0.137
9	Teamwork required in various fields	0.108	**0.711**	0.146	0.059
10	Importance of teamwork	0.163	**0.500**	0.053	0.129
8	Membership and leadership in group activities	0.128	**0.451**	0.092	0.014
1	Organization of the facility	0.150	0.152	**0.716**	0.086
2	Function of the facility	0.215	0.133	**0.701**	0.093
6	Your profession's role and uniqueness	0.316	0.207	0.103	**0.532**
	Department belonged to	-0.017	-0.019	-0.049	**-0.448**

*These data were analysed by the factor analysis method and the Kaiser's varimax method, by means of SPSS software.

### Subscale 1: Roles and responsibilities

The strongest item in the group was Q3 – "Roles of each profession in the facility" – with a factor loading of 0.781. This was followed by Q4, "Operations and tasks of each profession in the facility" (0.621) and Q5, "Collaboration among professionals working in the facility" (0.465).

### Subscale 2: Teamwork and collaboration

Three items were involved in this subscale. The strongest item in the group was Q9, "Teamwork required in various fields", with a factor loading of 0.711. This was followed by Q10, "Importance of teamwork" (0.500) and Q8, "Membership and leadership in group activities" (0.451).

### Subscale 3: Structure and function of training facilities

Two items contributed to this group with high scores. The first was Q1, "Organization of the facility" (0.716), and next was Q2, "Function of the facility" (0.701).

### Subscale 4: Professional identity

Two items contributed to this group, being related to positive and negative aspects of professional identity. The positively loaded item was Q6, "Your profession's role and uniqueness" (0.532), and the negatively loaded item was "Department belonged to" (-0.448).

### Comparison of mean scores on the survey for four health care students

**Figure 2:**
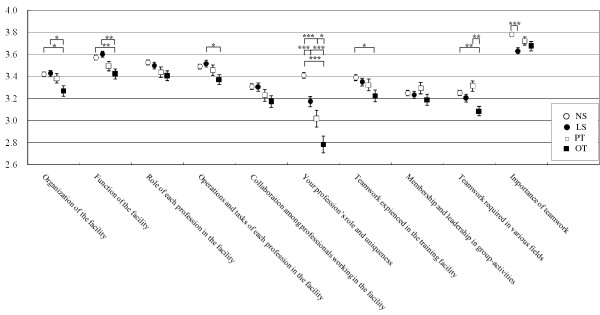
**Comparison of mean scores of each questionnaire in 4 health care departments**. One, two or three asterisks indicate significant difference, with p-value less than 0.05, 0.01 and 0.001, respectively.

In Figure 2, mean scores of individual items (Q1 – Q10) were compared for four health care students. Three out of ten items, Q3, Q5 and Q8, revealed no significant difference among four departments. In contrast, at Q6, "Your profession's role and uniqueness", there were significant differences among mean scores (mean and 95% confidence interval (95%CI)) of four departments (all p < 0.001), as follows: N 3.41 (95%CI, 3.36 – 3.46), LS 3.17 (95%CI, 3.08 – 3.26), PT 3.02 (95%CI, 2.87 – 3.16) and OT 2.78 (95%CI, 2.64 – 2.93).

It is noteworthy that in addition to this Q6, in five other items OT students also showed lower points as compared with other department students. The mean scores of OT department students for Q1, "Organization of the facility", and Q2, "Function of the facility", were significantly lower than those of NS and LS departments (p < 0.05). At Q9, "Teamwork required in various fields", the mean score of OT (3.09, 95%CI 3.00 – 3.17) was significantly lower than NS (3.25, 95%CI 3.21 – 3.30) (p < 0.01) and PT (3.31, 95%CI 3.22 – 3.41) (p < 0.01). For Q4, "Operations and tasks of each profession in the facility", there was a significant difference between OT (3.37, 95%CI 3.28 – 3.46) and LS (3.52, 95%CI 3.46 – 3.58) (p < 0.05). Also for Q7, "Teamwork experienced in the training facility", there was a significant difference between OT (3.22, 95%CI 3.12 – 3.33) and NS (3.39, 95%CI 3.34–3.44) (p < 0.05).

### Changes of the students' attainment over the years

**Figure 3:**
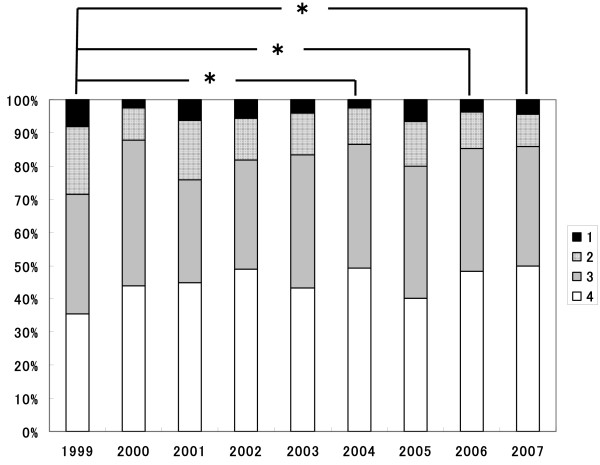
**Change of distribution of achievement to "Your profession's role and uniqueness" during 1999/2007**. White, gray, dotted or black sections indicate the percentage of students expressing "I fully understood", "I understood", "I did not understand well" and "I did not understand at all", respectively. The asterisk indicates significant difference with p-value less than 0.05.

The mean score of Q6, "Your profession's role and uniqueness" overall (3.22, 95%CI 2.37 – 7.07) was the lowest among 10 items and the scores of each department showed relatively heterogeneous distribution (Figure 2). The change of distribution of the attainment on Q6 during the nine years from 1999 to 2007 was investigated and the results are shown in Figure 3. The percentages of positive responses for "fully understand" and "understand" changed from 71.5% in 1999 to 86.0% in 2007. When mean scores of the surveyed year were compared by MANOVA model, the mean score and 95% CI in 1999 (2.99; 95%CI 2.84 – 3.14) was significantly lower than that in 2004(3.33; 95%CI 3.21 – 3.45), 2006 (3.30; 95%CI 3.17 – 3.43), and 2007 (3.32; 95%CI 3.19 – 3.44). The mean scores during 2000/2007 were not significantly different.

**Figure 4:**
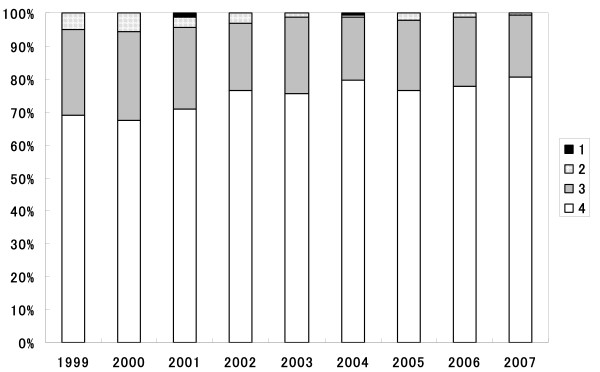
**Change of distribution of achievement to "Importance of teamwork" during 1999/2007**. White, gray, dotted or black sections indicate the percentage of students expressing "I fully understood", "I understood", "I did not understand well" and "I did not understand at all", respectively.

On the other hand, Q10, "Importance of teamwork" was the highest score of all the other nine items, and the scores of each department showed relatively homogeneous distribution (Figure 2). The mean scores were kept at a high level throughout the years examined (Figure 4), and were not significantly different during any of the periods evaluated.

### Open-ended opinions

Various opinions were obtained. Among them, requests for medical students to join the practice were expressed every year. For example, the statement was seen in 2006 as: "Collaboration with medical students is essential to achieve teamwork-training goals. Students of nursing, laboratory sciences and physical and occupational therapies would like medical students to join this training subject".

## Discussion

The mandatory IPE practice-training curriculum in GUSHS, designated as "Simulated interprofessional training among students of different professions in health sciences", has been implemented continuously for more than nine years. This educational programme was originally designed by us with two goals in mind: experiencing being part of a health care team (the spirit of interprofessional work), and learning interprofessional work in clinical practice (skills of interprofessional work).

Mandatory practice training has been assessed with original assessment tools. In the present study, four factor subscales – "Role and responsibilities", "Teamwork and collaboration", "Structure and function of training facilities" and "Professional identity" – were obtained when the questionnaire was analysed by factor analysis of the responses using varimax rotation to confirm the validity and reliability of questionnaires.

In 1999, Parcell & Bligh showed three subscales obtained from an IPE assessment using 19 items [5]. This assessment tool has been used widely for many types of IPE programmes; for example, in attitudes of health science faculty members towards IPE with minor modification [6]. It is of great interest that the present analyses reached the same three subscales as those described by Parcell & Bligh, i.e. "Role and responsibilities", "Teamwork and collaboration" and "Professional identity", although these two assessments were accomplished in completely different IPEs. These results suggest that the development of IPE programmes should require at least these three independent aspects.

On the other hand, our results included another subscale, "Structure and function of training facilities". The present IPE programme in GUSHS includes a two-day clinical training session at various types of facilities outside the university in the fields of medicine, health and welfare, while the assessment described by Parcell & Bligh did not include questionnaire items assessing these types of facilities [5]. These results suggest that the effectiveness and quality of the structure and functions at the facility may play an important role in the training-type IPE.

Surprisingly, the OT students showed lower points as compared with other department students in six items. The reason for the lower achievement of the IPE goals observed in OT students is uncertain at present. The lower comprehension of IPE in OT students might be unique to our university, since in an IPE initiative report by Johnston & Banks, no specific responses in OT students were described [7].

Recently, in a survey of 162 Bachelor of Health Sciences students, however, Hoffmann & Harnish reported a decrease in student interest in pursuing their professions after an IPE mandatory exercise by OT as well as nursing and social work students [8]. The significance of IPE with single professions, such as nursing [9] or a related profession [10], has been reported in OT students. This may be due to unique factor(s) in the introduction of IPE into OT student education; for example, a competitive professional situation (the rivalry among professions) reported in the United Kingdom [9] might exist. Alternatively, an active IPE in a clinical setting reportedly increased perceived collaborative and professional competence in four professions – MD, NS, PT, and OT; especially MD and OT students had the greatest gain [11].

In the present study, there was no significant decrease in the level of understanding in team-building questionnaire items – i.e., Q4, Q9, Q10 among students in the four departments. However, the questionnaire items regarding training facilities, Q1 and Q2, or professions' roles in the facilities, Q4 and Q7, showed a lower level of understanding in OT students. These results suggest that the achievement of IPE may depend on the training facility in OT students. Actually, occupational therapists worked in only nine out of the 19 training facilities in 2007.

Over 90% of respondents "really understood" or "understood" Q10 of "Importance of teamwork" and the mean score was the highest among all items. This high-level attainment was maintained throughout the nine years. On the other hand, the overall mean score of Q6 was the lowest. Interestingly, the mean score in the first year was significantly lower than that in 2004, 2006 and 2007, and the mean scores during 2000/2007 were not significantly different, indicating that the students' attainment of the present IPE programme in the first year was not sufficient as compared with the following years.

In Dalhousie University, the IPE initiatives by the School of Health Services Administration in the first year failed as well, while in the following year their interprofessional learning modules were successful [7]. These results may confirm the suggestion that an initiative of IPE should be kept and improved by increasing funding, participation by additional academic units such as medical departments, and the creation of a more formal management structure [4,7].

The desire for medical students to join in the training was voiced by many health profession students every year. When specific attributes of faculty members, including medicine, nursing, pharmacy and social work members, were examined in 2007, medicine faculty members reported significantly lower mean scores than nursing faculty on attitudes towards IPE [6]. For the successive delivery of IPE programmes, emphasis is placed on avoidance of stereotypes, enhancing communication and learning about the scope of practice of the different professions [10].

Furthermore, in some reports, the goals of the IPE initiatives seem to go beyond communication and role understanding, and suggest changing the culture of health professional interaction, referred to as flattening hierarchies [12]. Since higher-functioning teams are expected to have lower Physician Centrality scores [13], it has been implied that doctor authority may be detrimental to IPW [9]. However, it has been suggested that if collaboration depends on reducing doctor authority, it is unrealistic to expect that all doctors will readily be engaged in this process; conceptual models of teamwork and collaboration must articulate the desired nature of interaction between professionals with different degrees of responsibility and authority [9].

These findings suggest that development of a better understanding of how professional team members manage hierarchy and authority may play an important role in an effective health team. On the basis of the clinical settings, therefore, IPE may work well when students learn key communication strategies [3] resulting in successful patient outcome, such as family-based cardiovascular disease prevention and quality of care, postoperative pain and functioning, and length of stay in patients receiving total joint arthroplasty [2,14]. This process also provides an opportunity to think about how students recognize the authority in other professional departments before a "symbolic and psychological transformation" [15].

There are two main limitations of the present study. The first is the universality of the assessment tools used here. The present IPE programme has been planned, implemented and evaluated independently of scientific research. Comparative studies evaluating our original questionnaire to those used widely in the English-language literature [5,6] will be necessary, although the same three subscales were obtained from data using our original assessment tools, implying validity. Furthermore, the present subscales measure only "understanding", presumably a knowledge issue. An attempt to look at skills, attitudes, behaviour or performance will also be necessary. The second limitation is the lack of assessment of the role of two independent lecture-style IPE subjects implemented in the first academic year. This will be assessed carefully and elucidated.

## Conclusion

The effectiveness and the limitations of our unique assessment measures were statistically analysed over nine years (1999 – 2007). Our original questionnaire was categorized into four factors: "Role and responsibilities", "Teamwork and collaboration", "Structure and function of training facilities", and "Professional identity". All these factors play an essential role in the development of IPE training programmes.

We have started two main initiatives. One is participation by medical students, and the other is the introduction of simulated interprofessional training based on case scenarios into the group work before clinical training. These pilot initiatives should be assessed very carefully in the future. Also, clinical training subjects are remarkably dependent on the training facilities, as discussed in the lower assessment-points observed in OT students. We are developing a close consultative mechanism in the local network with the relevant educational institutes for medicine, health care and welfare in order to establish an ideal IPE, which will ideally lead to an IPW suitable for rural health care settings.

## Competing interests

The authors declare that they have no competing interests.

## Authors' contributions

HO, HT, TMak and HW participated mainly in the conception and design, and analysis. HO, TY and HW were those principally responsible for drafting this paper. All authors participated in the literature review, data collection and interpretation, and the final approval of the version of this manuscript to be published.

All members belong to the Interprofessional Education Committee of Gunma University (IPEC-GU).
